# Best *Helicobacter pylori* Eradication Strategy in the Era of Antibiotic Resistance

**DOI:** 10.3390/antibiotics9080436

**Published:** 2020-07-23

**Authors:** Su Young Kim, Jun-Won Chung

**Affiliations:** 1 Divison of Gastroenterology, Department of Internal Medicine, Yonsei University Wonju College of Medicine, 20 Ilsan-ro, Wonju 26426, Korea; breeze1212@naver.com; 2 Divison of Gastroenterology, Department of Internal Medicine, Gachon University, Gil Medical Center, 21, Namdong-daero 774beon-gil, Namdong-gu, Incheon 21565, Korea

**Keywords:** *Helicobacter pylori*, antibiotic resistance, eradication

## Abstract

Antibiotic resistance is the major reason for *Helicobacter pylori* treatment failure, and the increasing frequency of antibiotic resistance is a challenge for clinicians. Resistance to clarithromycin and metronidazole is a particular problem. The standard triple therapy (proton pump inhibitor, amoxicillin, and clarithromycin) is no longer appropriate as the first-line treatment in most areas. Recent guidelines for the treatment of *H. pylori* infection recommend a quadruple regimen (bismuth or non-bismuth) as the first-line therapy. This treatment strategy is effective for areas with high resistance to clarithromycin or metronidazole, but the resistance rate inevitably increases as a result of prolonged therapy with multiple antibiotics. Novel potassium-competitive acid blocker-based therapy may be effective, but the data are limited. Tailored therapy based on antimicrobial susceptibility test results is ideal. This review discussed the current important regimens for *H. pylori* treatment and the optimum *H. pylori* eradication strategy.

## 1. Introduction 

*Helicobacter pylori* is a Gram-negative, flagellated, spiral-shaped bacterium that penetrates the mucosal layer of the upper gastrointestinal tract [1,2]. It is responsible for peptic ulcers, gastric cancer, and other gastric diseases [3,4] and represents 25% of all infection-related malignancies; it is also associated with increased risk for gastrointestinal cancer [5]. Eradicating *H. pylori* relieves peptic ulcer disease and reduces the risk of gastric cancer [6,7]. Globally, the guidelines for the treatment of *H. pylori* are gradually changing, and the indications are expanding [4,8,9,10]. In addition, with the economic development of many developing countries and the increase in the aging population, the number of people receiving *H. pylori* eradication is increasing.

In the 1990s, triple therapy, consisting of a proton pump inhibitor (PPI), clarithromycin, and amoxicillin, was introduced for the eradication of *H. pylori* infection and is now the standard worldwide [11,12]. Since then, the eradication rate of triple therapy has markedly decreased in many regions [13,14]. The reasons for this decline include bacterial factors, reinfection, genetic polymorphisms of CYP2C19, and patient compliance [15]. Antibiotic resistance is the most important cause of failure of *H. pylori* treatment [13,16]. Resistance to clarithromycin of *H. pylori* is particularly serious; clarithromycin-containing regimens are no longer appropriate because of the <80% eradication rate [14,17]. The rate of resistance to metronidazole and quinolones (which are chiefly used as second- or third-line regimens) is >15% worldwide [14,16]. Because the antibiotics suitable for *H. pylori* eradication are limited (clarithromycin, amoxicillin, metronidazole, levofloxacin, tetracycline, and rifabutin), an increase in antibiotic resistance is a major problem. Therefore, the exclusion of clarithromycin is not enough to prevent antibiotic resistance of *H. pylori*. 

Current guidelines emphasize the importance of the local prevalence of antibiotic resistance when selecting a suitable *H. pylori* treatment regimen [4,9,10]. In areas with a high rate of resistance to certain antibiotics, treatment success can be improved by avoiding the use of such antibiotics. However, although a new combination of antibiotics may have an improved eradication rate, it may trigger resistance. The Taiwanese Government has restricted antimicrobial usage, resulting in a low rate of primary resistance to clarithromycin and metronidazole in *H. pylori* [18]. However, the rate of primary levofloxacin resistance increased from 4.9% in 2000–2007 to 13.4% in 2011–2012 [18]. In addition, the use of several antibiotics increases the rate of complications [4]. Therefore, it is important to determine the optimum *H. pylori* eradication strategy according to the results of antibacterial susceptibility testing (AST). However, the possibility of false negatives and applicability in all medical institutions are problems [19,20]. We reviewed the most important anti-*H. pylori* regimens, which overcome antibiotic resistance and strategies that can be applied in practice. 

## 2. *H. Pylori* Treatment Based on AST Results

Bacterial culture is typically performed before selecting an antibiotic, but *H. pylori* culture is intricate and time-consuming. Examination of the clarithromycin sensitivity of *H. pylori* improves the eradication rate [21,22]. Gerrits et al. showed using polymerase chain reaction (PCR) that the A2142G and A2143G mutations in 23S rRNA were associated with antibiotic resistance [22]. PCR is comparatively simple and cost-effective [21]. Therefore, for *H. pylori* treatment based on AST, such as a diagnostic test for clarithromycin resistance is useful. However, in some cases, the eradication rate is < 100%. This is because the point mutations other than A2142G or A2143G lead to unreliable results, e.g., A2142C, A2115G, G2142T, A2142T, and T2182C [23,24]. In addition, the distribution of mutations differs geographically. The A2142C mutation has an incidence of <10% in the United States and Europe but has not been reported in Japan [25,26,27,28]. In Hong Kong, the frequency of the A2144G mutation is higher than in other regions [29]. The different distributions of these mutations likely affect the eradication rate.

*H. pylori* therapy based on AST results significantly improves the eradication rate. However, unlike other regimens, few studies have evaluated AST-based first-line regimens. The intention-to-treat (ITT) eradication rate for a first-line regimen is >90% [30,31,32]. In South Korea, a PCR-based first-line tailored therapy is superior to the standard triple therapy (STT; amoxicillin, clarithromycin, and PPI) [30,31]. In a Greek study, genotypic resistance-guided triple therapy (clarithromycin and levofloxacin susceptibility testing by GenoType HelicoDR assay) has achieved a high *H. pylori* eradication rate (90.2% by ITT and 97.8% by per-protocol (PP) analyses) [32]. In addition, AST-guided first-line triple regimens result in a >90% eradication rate in patients with *H. pylori* resistant to two antibiotics [33]. Because the rate of multidrug-resistant *H. pylori* is increasing, this is an encouraging finding. Culture-based *H. pylori* first-line eradication regimens show an excellent therapeutic effect, even in regions with a high rate of antimicrobial resistance [34]. The overall resistance rates to amoxicillin, clarithromycin, metronidazole, and moxifloxacin are 6.7%, 31.0%, 41.8%, and 39.2%, respectively. However, the eradication rate is 93.1% (ITT) and 100.0% (PP) [34]. Chen et al. reported that a susceptibility-guided first-line modified bismuth quadruple regimen for *H. pylori* in a region with a high rate of antimicrobial resistance had a high eradication rate [35]. 

The success rate of AST-guided second-line/rescue regimens is lower than that of first-line regimens. The eradication rate is poor (68%) despite susceptibility testing for salvage treatment [36]. Likewise, in the United States, culture-guided therapy has shown a 60% success rate for patients who had failed at least three treatments [37]. Even if the culture identifies a clarithromycin-sensitive, rerunning clarithromycin after treatment failure is not recommended [38]. Therefore, AST-guided therapy alone cannot reach the required eradication rate. Liou et al. showed that genotypic tailored therapy was not significantly more effective than empirical therapy for rescue therapy strategy (78.0% vs. 72.2%, *p* = 0.170) [39]. Therefore, appropriate empiric therapy is an alternative to genotypic tailored therapy for refractory *H. pylori* infection.

## 3. Bismuth Quadruple Therapy 

Bismuth quadruple therapy (BQT) consists of bismuth, a PPI, metronidazole, and tetracycline. It is recommended as the first-line regimen by the Toronto Consensus, Maastricht V/Florence Consensus, and the American College of Gastroenterology (ACG) guidelines [4,9,10]. The BQT regimen is not affected by clarithromycin resistance. According to a network meta-analysis, BQT for 10 or 14 days is superior to STT for 7 days [40]. Moreover, BQT is highly effective as an empirical first-line regimen (PP and ITT eradication rates are 98.8% and 92.7%, respectively) [41]. A randomized controlled trial (RCT) in Taiwan yielded a 96.0% eradication rate in patients who received BQT, although the rate of adverse events was 47.7% [42]. BQT was highly effective as the first-line regimen for *H. pylori* eradication in a prospective study in Spain (94.4% (ITT) and 96.2% (PP)) [43]. BQT has an excellent *H. pylori* eradication rate, but patient compliance may be reduced because of the large number of drugs. In addition, BQT is administered four times daily, which may also reduce patient compliance. To overcome this, twice daily BQT regimens have been introduced, and studies in South Korea have demonstrated their effectiveness [44,45]. Kim et al. demonstrated that twice daily BQT for 1 week was as effective and safe as the conventional four times daily BQT (93.9% vs. 92.9%) [45]. In addition, most patients show good compliance, and the eradication rate of the twice-daily BQT is high (88.2% (ITT) and 98.4% (PP)) [44]. A single capsule containing bismuth, metronidazole, and tetracycline has been developed. Xie et al. showed that single-capsule BQT therapy was effective for *H. pylori* eradication and well-tolerated (86.5% (ITT) and 94.6% (PP)) [46]. In an Italian study, single-capsule BQT therapy achieved eradication rates of 91% (ITT) and 97% (PP) [47]. In a meta-analysis, first- and second-line single-capsule BQT therapy achieved an eradication rate approaching 90%. Even this applies, regardless of the type and dose of the PPI, in patients with antibiotic resistance strain and in those formerly treated with clarithromycin [48]. A 7-day BQT second-line regimen exhibits an eradication rate of 93.6% (PP). The eradication rate of 7-day BQT is significantly higher than that of 14-day moxifloxacin containing triple regimen (93.6% vs. 73.8% (PP), *p* < 0.001) [49]. 

Whether the eradication rate improves when the treatment period is extended from 7 to 14 days is unclear. The Maastricht V/Florence Consensus recommends administration for at least 10 days [10]. The rate of resistance to tetracycline is reportedly low worldwide [50,51]. Therefore, resistance to metronidazole is the primary determinant of the success of *H. pylori* eradication. Resistance to metronidazole can be overcome by increasing the frequency, amount, and duration of administration, so treatment for ≥10 days is recommended in areas with a high rate of metronidazole resistance [52]. 

BQT has been reported to have excellent results in various studies, and the scope of its application is gradually expanding. In addition, BQT can be used relatively safely in patients with penicillin allergy [4]. BQT reportedly has a higher rate of adverse events than STT but a similar rate of patient compliance [53,54]. However, as the indication of BQT expands as a first-line treatment, there are also concerns. The number of second-line regimens that can be applied is greatly reduced when eradication with BQT as the first line fails. In addition, the rate of resistance to tetracycline, which is at present relatively low, may increase in the future. Various studies are needed to overcome this problem in the future.

## 4. Concomitant Therapy

Concomitant therapy, three antibiotics (clarithromycin, metronidazole or nitroimidazole, and amoxicillin) and PPI administered concomitantly, is recommended for 10 to 14 days [4,9,10]. Several meta-analyses have shown that concomitant therapy is superior to STT [55,56,57]. In addition, a recent meta-analysis demonstrated that concomitant therapy for 5 or 10 days was superior to STT for 5, 7, or 10 days [58]. A Spanish study demonstrated that concomitant therapy was significantly better than triple therapy (92% vs. 70% (ITT), *p =* 0.02 and 92% vs. 74% (PP), *p* = 0.05), and the eradication rate of concomitant therapy was superior to that of sequential therapy for antibiotic-resistant strains [59]. In addition, concomitant therapy and BQT as first-line regimens have shown similar *H. pylori* eradication rates in PP (97.7% vs. 96.2%, *p* = 0.605) and ITT (98.0% vs. 94.4%, *p* = 0.346) analyses [43]. Moreover, network meta-analyses have shown that concomitant therapy has superior efficacy to several other regimens [40,60]. 

Concomitant therapy is preferred over sequential therapy (a PPI and amoxicillin for 5 days, followed by a PPI, clarithromycin, and tinidazole for another 5 days) because it is simpler. There is controversy over whether concomitant therapy is superior to sequential therapy [61,62,63,64,65]. In a Spanish RCT, concomitant therapy was non-significantly superior (~ 5%) to sequential therapy (87% vs. 81% (ITT), *p* = 0.15; 91% vs. 86% (PP), *p* = 0.131) [65]. In addition, two meta-analyses reported no significant difference in eradication rate between concomitant therapy and sequential therapy [66,67]. 

The Maastricht V/Florence consensus does not recommend sequential therapy, unlike previous guidelines [10]. This is because sequential therapy has a lower eradication rate than concomitant therapy in cases of clarithromycin-resistant and metronidazole-susceptible *H. pylori* strains. Conversely, when *H. pylori* is susceptible to clarithromycin and resistant to metronidazole, sequential therapy shows a lower eradication rate than STT [10]. Increasing the dose or duration of metronidazole treatment may lead to the eradication of metronidazole-resistant *H. pylori*. The eradication rate of sequential therapy is low because the period of metronidazole administration is only 5 days.

Concomitant therapy has several limitations, such as a higher rate of adverse events than sequential therapy [68]. An increased frequency of complications may affect compliance with *H. pylori* treatment [69,70]. Although the frequency of adverse events is relatively high, the treatment period is <2 weeks, so the majority of patients complete the treatment course. In addition, there is concern that antibiotic resistance may be increased by excessive exposure to unnecessary antibiotics [71]. Finally, the effects of concomitant therapy are lower in *H*. *pylori,* resistant to both clarithromycin and metronidazole [72]. 

## 5. Hybrid Therapy

Hybrid therapy is a combination of sequential and concomitant therapy. Hybrid therapy comprises a PPI and amoxicillin for 7 days, followed by a PPI, amoxicillin, clarithromycin, and metronidazole for 7 days [4]. Although the Toronto Consensus and Maastricht V/Florence Consensus do not recommend hybrid therapy, the ACG clinical guidelines recommend its use as a first-line treatment in patients without prior macrolide exposure in regions with a low rate of clarithromycin resistance. Several meta-analyses have reported the efficacy and tolerability of hybrid therapy [40,67,73]. Wang et al. demonstrated that hybrid therapy was an alternative to concomitant or sequential therapy (ITT eradication rates of hybrid, concomitant, and sequential therapy were 88.6%, 86.3%, and 84.7%, respectively; PP eradication rates were 92.1%, 92.5%, and 87.5%) [73]. In addition, there are no significant differences in tolerability or compliance between hybrid therapy and STT, sequential, or concomitant regimens [40,67,73]. 

In a prospective multicenter study, hybrid therapy cured >90% of patients with *H. pylori* infection in areas with high rates of clarithromycin and metronidazole resistance; this is similar to the eradication rate of concomitant therapy [74]. In addition, compliance with hybrid therapy is superior to that of concomitant therapy (98.8% vs. 95.2%, *p =* 0.05). However, the eradication rate of hybrid therapy is significantly lower for dual-resistant *H. pylori*. In a prospective study, the eradication rate in patients with dual antibiotic resistance (clarithromycin and metronidazole) was noticeably decreased (50%) compared to those with only clarithromycin resistance (91.4%) or metronidazole (90.5%) resistance [75]. 

## 6. Levofloxacin-Based Therapy

Levofloxacin is active on a large spectrum of various bacteria, and some studies use levofloxacin as the first-line treatment of *H. pylori* infection [15]. A prospective study in China showed that cefuroxime, levofloxacin, a PPI, and bismuth as first-line therapy achieved an *H. pylori* eradication rate of 97.2% of levofloxacin-susceptible cases and 84.0% of levofloxacin-resistant cases [76]. However, the eradication rate has been 0% in cases of resistance to both cefuroxime and levofloxacin. Once-daily 14- and 7-day levofloxacin dosing regimens (levofloxacin, clarithromycin, rabeprazole, and bismuth) have shown eradication rates of 94% and 84%, respectively [77]. Gan et al. compared the efficacy of different dosages of levofloxacin for the eradication of *H. pylori* [78]. The eradication rates in the once- and twice-daily groups were 77.5% and 79.5% (ITT) and 82.9% and 86.4% (PP), respectively [78]. There are no significant differences in the eradication or compliance rate. Bovine lactoferrin enhances the efficacy of levofloxacin-based first-line regimens for *H. pylori* infection [79]. The eradication success rate is 96.1% for esomeprazole/amoxicillin/levofloxacin/bovine lactoferrin and 75% for esomeprazole/amoxicillin/levofloxacin [79]. The LOAD (levofloxacin, omeprazole, nitazoxanide, and doxycycline) regimen has been introduced recently [71]. Basu et al. reported that LOAD had an eradication rate of 89.4% compared to 73.3% for STT (*p* < 0.05) [80]. 

Levofloxacin is a quinolone and is widely used worldwide for, for instance, pneumonia, urinary tract infection, tuberculosis, and *H. pylori*. Therefore, many patients have a history of exposure to levofloxacin. Most guidelines recommend that levofloxacin-based regimens be applied as a rescue rather than a first-line therapy [81]. The ACG clinical guidelines weakly recommend levofloxacin-based triple (amoxicillin, levofloxacin, and PPI), sequential (5 to 7 days of a PPI and amoxicillin, followed by 5 to 7 days of a PPI, nitroimidazole, and levofloxacin), or quadruple (a PPI, levofloxacin, doxycycline, and nitazoxanide for 7 or 10 days) first-line regimens [4]. 

Sitafloxacin is a fourth-generation fluoroquinolone and has potent activity against *gyr*A mutation-positive *H. pylori* strains [82]. Sitafloxacin is mainly used with amoxicillin or metronidazole as a rescue rather than first-line therapy. Sitafloxacin-containing third-line regimens are reportedly effective for *H. pylori* eradication (75.8% (ITT) and 83.3% (PP)) [83]. Fourth-generation fluoroquinolones, such as sitafloxacin and garenoxacin, may overcome the quinolone resistance of *H. pylori* [84,85]. Among 100 strains with high rates of resistance to clarithromycin, metronidazole, and levofloxacin, >95% are susceptible to sitafloxacin [86]. The efficacy of sitafloxacin or garenoxacin-containing regimens against *gyr*A mutation-positive *H. pylori* should be evaluated.

## 7. Rifabutin-Based Therapy

Rifabutin is a rifamycin derivative and structurally similar to rifampicin (anti-tuberculosis drug). It is mainly used for atypical tuberculosis, such as *Mycobacterium avium*-intracellulare and *M. tuberculosis* resistant to rifampicin [87]. Rifabutin suppresses protein synthesis by inhibiting the beta-subunit of the *Helicobacter* DNA-dependent RNA polymerase, which is encoded by *rpoB*. Therefore, mutation of *rpoB* confers resistance to rifabutin [88]. All extant guidelines suggest that rifabutin-based regimens be considered only as rescue therapies for *H. pylori* eradication [81]. According to a meta-analysis, the cure rates of the second-, third-, and fourth/fifth-line rifabutin-based therapies are 79%, 66%, and 70%, respectively [87]. In an Italian study, rifabutin-based triple therapy (rifabutin, amoxicillin, and PPI) for 14 days achieved an eradication rate of 72.7% (PP) and 71.5% (ITT) in patients in whom *H. pylori* eradication failed following treatment with conventional antibiotics [89]. Rifabutin does not share antibiotic resistance with clarithromycin and amoxicillin and is fat-soluble and readily absorbed after oral intake [88,90]. In addition, rifabutin is stable at a wide range of pH values and is not degraded by gastric acid, and the rate of resistance in *H. pylori* is low because it is rarely used clinically [87,91]. Therefore, rifabutin-based regimens are important for rescue therapy. However, another study demonstrated that the eradication rate of rifabutin-based rescue therapy was not good, with 50.0% in ITT analysis and 54.5% in PP analysis [92]. As there are some parts that do not achieve a stable eradication rate as rescue therapy, it may be considered that related studies are needed. 

To date, most studies on rifabutin-based regimens have focused on rescue therapy. Recently Graham et al. showed that a rifabutin-based triple regimen (amoxicillin, rifabutin, and a PPI) had a higher eradication rate than amoxicillin plus a PPI as the first-line empirical treatment of *H. pylori* (83.8% vs. 57.7%, *p* < 0.001) [93]. This suggests that rifabutin is a breakthrough first-line treatment in the era of antibiotic resistance. However, it is more expensive than other antibiotics. In addition, it may induce resistance to the regimen of tuberculosis treatment [94]. Moreover, because the use of rifampicin promotes point mutation of *rpoB* and increases the minimum inhibitory concentration (MIC) of rifabutin, previous rifampicin treatment must be determined before administering rifabutin [95]. Finally, rifabutin has a small risk for myelotoxicity [87]. 

## 8. Potassium-Competitive Acid Blocker-Based Therapy

Potassium-competitive acid blocker (P-CAB) is an inhibitor of gastric acid secretion, with a faster onset and longer-acting acid suppression, and is more potent than a PPI [96,97]. In addition, P-CAB does not require activation by stomach acid, so it can be taken irrespective of the timing of meals. In the treatment of *H*. *pylori,* gastric pH must be maintained above a certain level to enable antibiotic activity [98]. P-CAB inhibits H^+^ and K^+^-ATPase-mediated gastric acid secretion and is acid-stable and less impaired by the CYP2C19 system than PPIs [71,99]. Because PPIs have a short duration of action and their efficacy is influenced by various subtypes of cytochrome P450 (PPIs are mainly metabolized by CYP2C19 and CYP3A4), a new anti-*H. pylori* regimen containing PCAB has attracted attention [99,100]. Vonoprazan is currently marketed in Japan, promoting research on P-CAB for *H. pylori* treatment.

According to a multicenter RCT in Japan, the first-line eradication rate of vonoprazan-based triple therapy (vonoprazan, amoxicillin, and clarithromycin) was 92.6% vs. 75.9% for STT (*p* < 0.0001) [101]. A meta-analysis of Japanese studies involving 1599 patients demonstrated that vonoprazan-based triple therapy was significantly superior to STT for patients with clarithromycin-resistant strains (82.0% vs. 40.0%, *p* < 0.0001) [102]. By contrast, the eradication rate of clarithromycin-susceptible strains is not significantly different. If the organism is resistant to clarithromycin, the STT is slipped to the dual therapy (PPI + amoxicillin), and dual therapy is important to maintain gastric pH at ≥6. In this regard, it is thought that vonoprazan has strengths. In addition, a vonoprazan-based triple regimen is as effective as susceptibility-guided PPI-based STT for *H. pylori* eradication (97.4% vs. 95.7%) [103]. In a study of the efficacy of 7-day vonoprazan and low-dose amoxicillin dual therapy as a first-line *H. pylori* treatment, the eradication rates of dual (vonoprazan and low-dose amoxicillin) and triple therapies (vonoprazan, low-does amoxicillin, and clarithromycin) were 84.5% and 89.2% (*p* = 0.203) by ITT analysis, respectively, and 87.1% and 90.2% (*p* = 0.372) by PP analysis, respectively [104]. In the reality of increasing antimicrobial resistance, it can be said that the decrease in the total amount of antibiotics using vonoprazan shows a positive aspect in the future treatment of *H. pylori*. 

In Japan, P-CAB-based regimens are licensed as first- and second-line treatment for *H. pylori* eradication. Murakami et al. demonstrated the efficacy of a P-CAB-based second-line triple regimen (success rate of 98.0%) [101]. Another RCT showed that vonoprazan-based triple therapy (vonoprazan, amoxicillin, and sitafloxacin) was more effective when PPI was used with those antibiotics as a third-line treatment regimen for *H. pylori* (83.3% vs. 57.1% (PP), *p* = 0.043) [83]. Therefore, P-CAB-based therapy may be a viable alternative first- and second-line regimen for *H. pylori* eradication.

## 9. Treatments When Primary Therapy Fails

All guidelines recommend avoiding antibiotics taken by a patient previously [4,8,9,10]. In particular, reuse of clarithromycin and levofloxacin must be avoided because of the high rates of resistance. They also recommend a BQT- or levofloxacin-based triple second-line treatment regimen for *H. pylori* [81]. Susceptibility testing for appropriate rescue therapy should be considered if the first- or second-line therapy fails [10,81]. 

According to the Maastricht V/Florence Consensus, after the failure of BQT as first-line treatment, a quinolone-containing triple or quadruple therapy is recommended [10]. After the failure of a first-line non-bismuth quadruple regimen, a BQT- or quinolone-containing triple or quadruple regimen is recommended [10]. BQT, non-bismuth quadruple therapy, and quinolone-containing triple therapy can be used in a non-overlapping manner with the second- or third-line treatment regimen [10]. 

The Toronto Consensus and the ACG guidelines, like the Maastricht V/Florence Consensus, emphasize the importance of BQT and levofloxacin-containing regimens as second-line [4,9]. An important difference from the Maastricht V/Florence Consensus is the opposition to the use of non-bismuth quadruple second-line regimen because this is less efficacious than other therapies [9]. In addition, rifabutin-based regimens should be reserved for patients with at least three previous treatment failures [9]. The ACG guidelines state that the second-line regimen should be selected based on previous exposure to antibiotics and local antibiotic resistance data [4].

## 10. Conclusions

**Figure 1:**
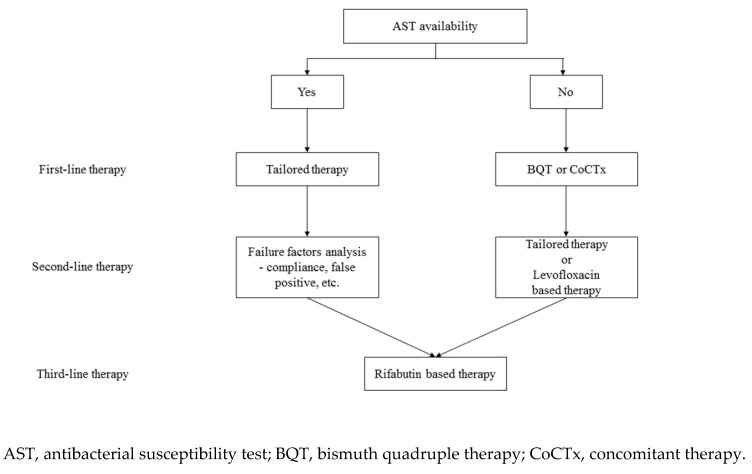
Simplified *H. pylori* treatment strategy.

Table 1 summarizes important regimens for the treatment of *H. pylori*. *H. pylori* treatment is an important global issue because it has been directly implicated as a cause of several gastrointestinal diseases. There is clear concern about increased rates of resistance over time, highlighting the necessity for suitable antibiotic use going forward to minimize further growth of antibiotic resistance. AST-based tailored therapy showed a good eradication rate when applied first-line. Because the rate of antibiotic resistance of *H. pylori* is increasing, the role of tailored therapies should be expanded. However, if tailored therapy cannot be applied to all patients due to realistic problems, using a locally highly effective empiric regimen is a reasonable alternative. In addition, when using such an empiric regimen, a simple and efficient *H. pylori* treatment strategy is needed. Therefore, we suggest BQT as first-line therapy when AST or regional resistance data are not available (Figure 1). If BQT is unavailable, we suggest empiric concomitant therapy. P-CAB may be an alternative, but data are sparse and restricted to East Asia.

Tailored therapy, according to the AST results, promotes *H. pylori* treatment without increasing antibiotic resistance, so susceptibility testing should be emphasized. There are also additional considerations; for example, how to set the duration of treatment if susceptible to clarithromycin and how to choose a regimen (BQT or amoxicillin-metronidazole-PPI) if resistance to clarithromycin has not been studied. Further work should focus on maximizing the therapeutic effects of tailored therapies based on the results of AST.

## Figures and Tables

**Table 1:** Regimens for the treatment of *Helicobacter pylori* infection.

Treatment	Regimen	Duration	Recent First-Line Eradication Rate (ITT)	Recommendations According to Guidelines	Notes
Standard triple therapy (STT)	PPI standard dose bid Amoxicillin 1 g bid Clarithromycin 500 mg bid	7–14 d	63.9% [105], 74.1% [106]	First-line: optionally recommended by KCHUGR and JSHR Rescue: limited recommended by MAA	Over the past 20 years, the efficacy of STT has markedly decreased, and STT is generally not recommended as a first-line regimen
Bismuth quadruple therapy (BQT)	PPI standard dose bid Bismuth standard dose qid Metronidazole 500 mg tid Tetracycline 500 mg qid	7–14 d	82.8% [107], 88.2% [44], 91.5% [108]	First-line: recommended by ACG, MAA, TOR, and KCHUGR (optionally) Rescue: recommended by ACG, MAA, TOR, and KCHUGR	BQT has been suggested as a first-line treatment option in many guidelines, especially for regions with a high clarithromycin resistance.
Concomitant therapy (non-bismuth quadruple therapy)	PPI standard dose bid Clarithromycin 500 mg bid Amoxicillin 1g bid Metronidazole 500 mg bid	10–14 d	84.6% [106], 90.1% [109], 93.5% [110]	First-line: recommended by ACG, MAA, and TOR Rescue: recommended by ACG and MAA	The eradication rate is superior to that of CTT, and the method of administration is simple compared to that of sequential therapy. But, adverse events may be more likely with concomitant therapy.
Sequential therapy	PPI standard dose bid Amoxicillin 1g bid (first half only) Clarithromycin 500 mg bid (for the second half only) Metronidazole 500 mg bid (for the second half only)	10–14 d	69.5% [106], 82.0% [111], 87.0% [112]	First-line: optionally recommended (not ideal) by ACG Rescue: not recommended in all guidelines	As first-line therapy, the role is gradually disappearing. It is a cumbersome way to reduce patient compliance.
Hybrid therapy	PPI standard dose bid Amoxicillin 1g bid Clarithromycin 500 mg bid (for the second half only) Metronidazole 500 mg bid (for the second half only)	14 d	85.8 % [75], 92.8% [113]	First-line: optionally recommended (not ideal) by ACG Rescue: not recommended in all guidelines	It is a method that combines sequential therapy and concomitant therapy.
Levofloxacin-based therapy	Levofloxacin can be given as triple therapy or quadruple therapy.	10–14 d	85.5% [76], 94.0% [77]	First-line: recommended by ACG Rescue: recommended by ACG, MAA, and TOR	Most guidelines recommend that levofloxacin-based therapy be applied as rescue therapy rather than first-line. It is less effective for areas with high quinolone resistance.
Rifabutin-based therapy	PPI standard dose bid Amoxicillin 1g bid Rifabutin 150 mg bid	10 d	83.8% [93]	First-line: not recommended in all guidelines Rescue: optionally recommended (third or fourth-line) by MAA and TOR	All guidelines recommend rifabutin-based therapy as rescue therapy. Rifabutin has the rare risk of myelotoxicity; therefore, careful use is required.
Potassium-competitive acid blocker based therapy	P-CAB can be given as triple therapy or quadruple therapy by replacing PPI with P-CAB.	7–14 d	89.2% [104], 90.2% [114]	Not stated in algorithm of guidelines	The role of potent acid suppression is expected to increase gradually, and more research is needed.
*H. pylori* treatment based on antibacterial susceptibility test	Tailored therapy according to AST results	7–14 d	92.7% [31], 92.9% [115]	MAA recommends to perform AST after the failure of second-line treatment.	The results of tailored therapy based on AST are excellent, and it is expected to play a role in improving *H. pylori* treatment in the future. Efforts to facilitate the application of AST in clinical practice are required.

ITT, intention to treat; STT, standard triple therapy; PPI, proton pump inhibitor; KCHUGR, Korean College of Helicobacter and Upper Gastrointestinal Research [116]; JSHR, Japanese Society for Helicobacter Research [117]; MAA, Maastricht V/Florence Consensus [10]; BQT, bismuth quadruple therapy; ACG, American College of Gastroenterology clinical guideline [4]; TOR, Toronto Consensus [9]; CTT, concomitant therapy; P-CAB, potassium-competitive acid blocker; AST, antimicrobial susceptibility test.
